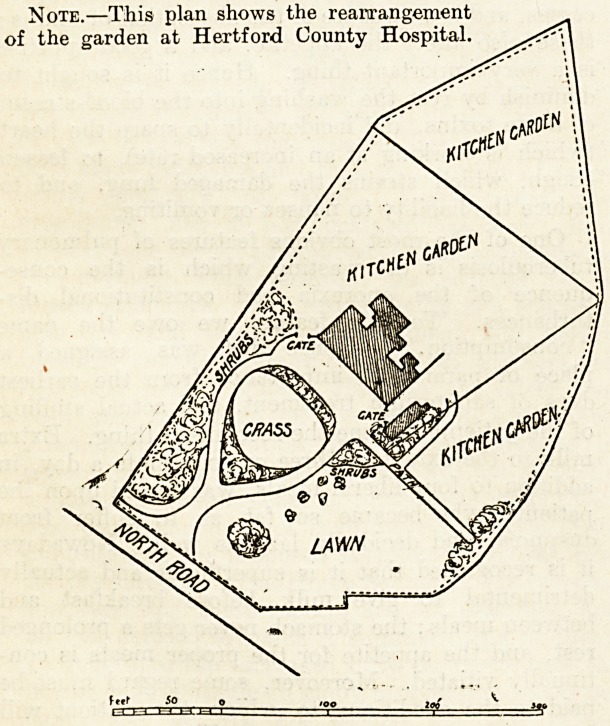# Remodelling a Hospital Garden

**Published:** 1916-05-20

**Authors:** 


### REMODELLING A HOSPITAL GARDEN.

### A Practical Example at Hertford.

Before the war The Hospital published a series of Reports upon various German hospitals. Setting aside, for the moment, certain grave points (that patients were sacrificed to "science," for instance) on which criticism was compelled to fasten, praise was given to many of the hospital gardens which are a frequent feature of institutional planning abroad. Hospital gardening in the United Kingdom is in its infancy. Occasionally a little bedding-out is done, or a piece of vegetable garden is developed; but, even if we remember the attempts made in the grounds of mental hospitals, hospital gardening is certainly not regarded as an integral part of hospital design. Believing that in hospital gardening, at all events, we have something to learn from the Continental institutions, every attempt, even on a small scale, deserves some notice and encouragement.

Note.—This plan shows the rearrangement of the garden at Hertford County Hospital.

Thus, at the Hertford County Hospital, the acquisition of a piece of land to the right of the entrance has led to a consideration of the best way in which the hospital grounds might be improved. Mr. George Paul, of the Old Nurseries, Cheshunt, was consulted, and the point which he was asked to decide was whether it was desirable entirely to rearrange the ground in front of the hospital, or slightly to alter the existing plan.

The rough sketch which is appended makes the position clear. Since the new land on the right of the entrance very largely extended the frontage of the hospital, it would have been possible, if desired, to use the land so as to form a really elaborate approach to the hospital. There was relatively plenty of space "to play about with." But for the sake of economy, and because the existing plan might be modified without much difficulty, Mr. Paul decided to adapt the new land to the right of the entrance and fit it in with the central lawn.

It was not, then, a case for landscape gardening, except in the sense that this part of the hospital could be very much improved, not only in appearance, but for the benefit of the patients.

The gravel front was first of all widened, and, with the lawn, a large space was therefore provided for the convalescents to walk about in, with an extended walk ending at a seat near the boundary of the property. It so happened that the kitchen garden, which, as appears from the plan, occupies a large part of the site, is already intersected with paths, and in the past the convalescents have taken advantage of them. By the new plan they are much better provided for in this respect, and, as the frontage of the hospital has been much improved. the change is an interesting example of how much can be done on the right lines, even in a small way.

## Figures and Tables

**Figure f1:**